# New insights into DMD using mouse and canine models

**Published:** 2013-09

Two research articles published in the current issue progress our understanding of Duchenne muscular dystrophy (DMD), a debilitating X-linked disorder characterised by progressive muscular weakness and degeneration. The disorder is caused by mutations in the gene encoding dystrophin, an important component of the dystrophin-glycoprotein complex (DGC) that connects intracellular proteins with the extracellular matrix and promotes muscle fibre integrity. In the first of the two studies, Mariz Vainzof and colleagues report a new mouse model for the disease in the form of a *Dmd^mdx^*/*Large^myd^* double mutant (**page 1167**). In addition to lacking dystrophin, this mouse also expresses a mutated version of the gene encoding LARGE, another important component of the DGC. The combination of these two mutations provides a model that demonstrates a severe neuromuscular phenotype and accurately mimics the symptoms of human muscular dystrophies. The authors also demonstrate that the model could be used to test the benefit of stem cell therapy to treat DMD and associated disorders. In a second study, Dean Burkin’s group provides insight into the mechanism underlying the beneficial effects of corticosteroid therapy in DMD and reveal potential candidates to target in future therapeutic approaches that might overcome the negative side effects associated with steroid use (**page 1175**). In their work, they utilise *in vitro* cultured myogenic cells and also a canine model of DMD that shows strong phenotypic similarities to human DMD patients, demonstrating that the use of multiple complementary approaches and model systems can allow complex disease mechanisms to be elucidated.

**Figure:**
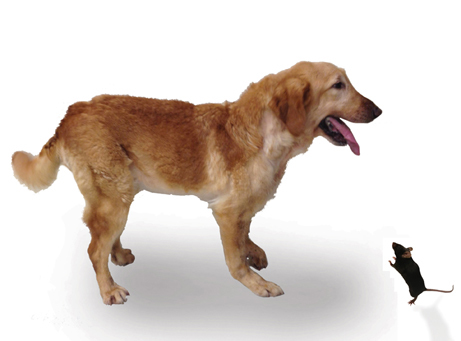
Dean Burkin’s group thank Scott Barnett, Department of Pharmacology, University of Nevada, Reno, NV, for his assistance in producing the image.

